# Monitoring Fish Biodiversity in the Pelagic Zone of the Western Indian Ocean Using Environmental DNA Metabarcoding

**DOI:** 10.3390/biology14091194

**Published:** 2025-09-04

**Authors:** Ding Lyu, Rihong Xu, Yue Jin, Yulong Hu, Mianyu Liu, Guanzheng Lyu, Xiujuan Shan, Weiji Wang

**Affiliations:** 1 State Key Laboratory of Mariculture Biobreeding and Sustainable Goods, Yellow Sea Fisheries Research Institute, Chinese Academy of Fishery Sciences, Qingdao 266071, China; lvding@ysfri.ac.cn (D.L.); xu15853264926@126.com (R.X.); jinyue@ysfri.ac.cn (Y.J.); yulonghu163@163.com (Y.H.); 2022213007@stu.njau.edu.cn (M.L.); 14717669732@163.com (G.L.); 2 Laboratory for Marine Fisheries Science and Food Production Processes, Laoshan Laboratory, Qingdao 266237, China; 3 College of Fisheries, Nanjing Agricultural University, Nanjing 210095, China

**Keywords:** species diversity, Indian Ocean, environmental DNA, pelagic zone, fish

## Abstract

The Indian Ocean is globally significant in terms of capture fisheries, and understanding the species composition of fish in the Indian Ocean is of great importance for the protection and development of its fishery resources. While coastal fish communities in the Indian Ocean are relatively well-documented, studies on pelagic zones remain sparse, especially for non-target species constituting fishery bycatch. Traditional biodiversity surveys rely on labor-intensive, inefficient trawling methods. To address these limitations, this study aims to apply environmental DNA (eDNA) metabarcoding for a species diversity survey in the Western Indian Ocean, offering a more reliable, efficient, and non-invasive alternative to traditional methods. The results will provide important insights into the region’s fish biodiversity, supporting sustainable management and conservation of fisheries resources in the area. Samples were collected from 130 stations in different water layers in the Western Indian Ocean, and species diversity was analyzed through 12S rRNA gene amplicon sequencing. The results showed that 98 fish species were detected from 176 seawater eDNA samples, belonging to two classes (Actinopteri and Chondrichthyes), 20 orders, 35 families, and 60 genera. Within a depth range of 300 m, there were no significant differences in species diversity parameters among samples from different depths. The orders with the highest relative abundance detected include Scombriformes, Aulopiformes, and Myctophiformes. The species with the highest relative abundance include *Thunnus albacares*, *Alepisaurus ferox*, *Xiphias gladius*, *Diaphus fragilis*, *Decapterus macarellus*, *Thunnus maccoyii*, and *Platycephalus cultellatus*. The species composition and relative abundance of economic species observed in this study showed, as expected, differences from fishery catch statistics. These results suggest that eDNA technology can not only monitor marine fish diversity more efficiently but also complement the lack of fisheries data. Integrating eDNA technology into routine monitoring in the Western Indian Ocean in the future could promote sustainable management of fisheries resources in the region.

## Simple Summary

Knowing what kinds of fish live in the open waters of the Indian Ocean is important for protecting fish stocks and making sure fishing is sustainable. However, it is difficult and time-consuming to study these fish using traditional methods like nets, especially for fish not directly targeted by fishermen. This study tested a new method using environmental DNA in the Western Indian Ocean. Instead of catching fish, scientists collected seawater from different depths at 130 locations and looked for tiny traces of DNA that fish leave behind. By reading this DNA, they identified 98 different fish species living in the area, including types like tuna, swordfish, and lanternfish. They found that the variety of fish detected was similar down to about 300 m deep. The results also showed that this DNA method gives a different picture of which fish are common compared to what fishermen usually catch. This study shows that eDNA is a reliable and less harmful way to monitor ocean fish life. Using this method regularly in the future can help scientists and managers obtain a more complete understanding of fish populations in the Western Indian Ocean, leading to better decisions for protecting these vital resources and ensuring long-term sustainable fishing.

## 1. Introduction

The Indian Ocean is the world’s third-largest ocean, covering approximately 29% of the global ocean area [1], and its fisheries catch accounts for about 15% of the global marine capture production [2]. While catch volumes in other major ocean regions are declining, the Indian Ocean remains one of the few areas where fishing yields have continued to grow [1,2]. Understanding the fish composition of the Indian Ocean is crucial for assessing the socio-economic impact on the region, as fish are a primary source of food and income for many coastal communities [3]. At the same time, changes in the fish composition of the Indian Ocean can reflect the impact of climate change on marine ecosystems, providing valuable information for predicting future trends and developing sustainable management strategies [4]. Biodiversity surveys of fishery resources generally rely on trawling methods, which are laborious, costly, time-consuming, and have low sampling efficiency. These methods are also susceptible to external factors such as water conditions and fishery policies, which can make surveys less reliable [5,6,7]. Currently, data on fishery resources in the Indian Ocean primarily rely on commercial fishing records; however, due to limited research depth and being predominantly sourced from coastal fisheries, these data may not provide comprehensive or accurate information [8]. In addition, whether trawling surveys or obtaining data from commercial fishing, these capture-based sampling methods rely on subsequent morphological identification of specimens based on taxonomic expertise [9,10].

In order to complement traditional methods, environmental DNA (eDNA) metabarcoding has emerged in recent years and played an increasingly important role in the investigation of fishery resources [11,12]. eDNA consists of the DNA molecules or fragments that organisms continuously shed into the environment through biological materials such as skin, mucus, saliva, sperm, eggs, feces, urine, and decomposing bodies [13]. The technology associated with eDNA involves the qualitative and quantitative analysis of DNA extracted from environmental samples. This is achieved using techniques like quantitative PCR and high-throughput sequencing, enabling researchers to infer and assess the presence, species diversity, abundance, and distribution of organisms within a specific environment [14,15,16]. Compared to traditional monitoring methods, eDNA analysis offers several advantages, including its non-invasive nature, high sensitivity, and the ability to detect multiple species simultaneously [17].

In the field of fish eDNA research, Miya et al. designed a pair of universal primers for the mitochondrial 12S rRNA gene partial sequence (163–185 bp) of marine fishes [18]. Combined with high-throughput sequencing, PCR amplification with this primer pair can identify multiple marine teleost species simultaneously. With the help of this technology, 168 species were identified from 180 species of marine fish in the aquarium environment [18]. In a marine fishery resources survey, Stoeckle et al. employed the eDNA method to survey fish resources along the New Jersey coast, USA [19]. They found high consistency between bottom trawling and eDNA methods in detecting resource abundance, fish composition, and seasonal distribution. During monthly surveys, between 70% and 87% of the species detected by trawling were also identified using eDNA, while 92% to 100% of the species detected by eDNA were also found through trawling [19]. In a previous study by our team, we applied eDNA metabarcoding to analyze fish species diversity and relative abundance in the Yellow River estuary. The results revealed 33 species detected by eDNA, compared to 31 species identified by trawl surveys, with 21 species detected by both methods. For these 21 species, there was a high correlation between eDNA relative abundance and catch per unit effort (CPUE) [20]. These findings, along with other studies, have confirmed the potential of eDNA metabarcoding in fishery resource assessment [7,9,19,20,21].

In this study, we aim to conduct a species diversity survey in the Indian Ocean using eDNA. We collected water samples from 130 stations across three different water layers in the Western Indian Ocean and analyzed eDNA from these water samples to explore the composition of fish species in this region. While eDNA has been applied in coastal waters globally, this represents the first systematic application for pelagic fish monitoring in the Western Indian Ocean, a region of critical importance for global fisheries. The results of this research should complement traditional fish surveys based on catch data and provide valuable insights for the sustainable management of fisheries resources in the Western Indian Ocean.

## 2. Materials and Methods

### 2.1. Study Area and Sample Collection

**Figure 1:**
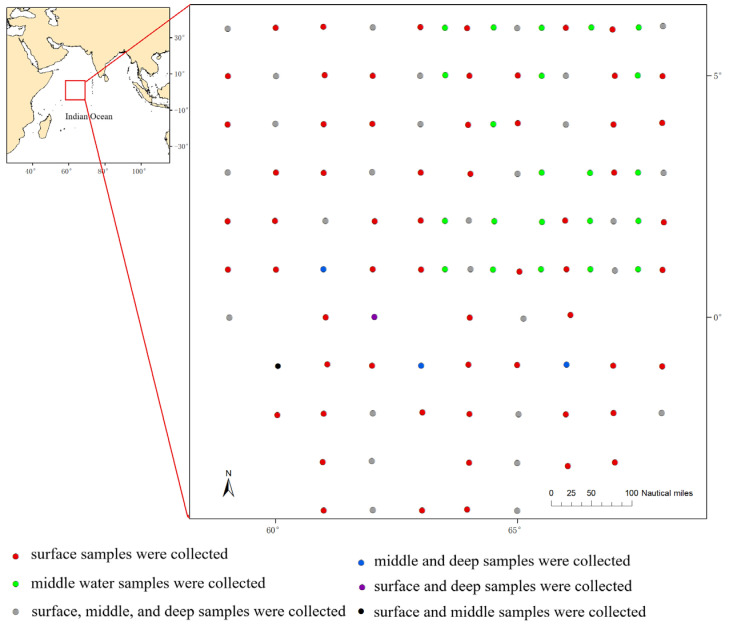
Distribution of water sampling stations in the Western Indian Ocean.

We conducted water sampling from 14 December 2023 to 2 February 2024. The survey area covered the Western Indian Ocean (58° E–68° E, 5° S–6° N), with a total of 130 survey stations (Figure 1). The total coverage area of all station positions is approximately 396,000 nmi^2^, with adjacent stations spaced about 15 nmi apart. Out of the 130 stations, 34 stations were sampled for surface (with the depth of 0 m), middle layer (100 m), and deep (300 m) water samples; 66 stations were sampled only for surface water; and 30 stations were sampled only for middle layer water. Thus, a total of 198 water samples were collected. Due to the large sample size in this study, sample duplication was not set in this study.

We collected water samples from different layers using the SBE-911 plus CTD (Sea Bird Scientific, Bellevue, WA, USA). After collection, samples were filtered with a 0.7 μm Glass Microfiber Filter (Whatman, Maidstone, Kent, UK) and a diaphragm vacuum pump (Jinteng, Tianjin, China). Each sample was filtered with a volume of 1 L. The SBE-911 plus CTD was repeatedly rinsed with deionized water. Filter flasks and forceps were disinfected with sodium hypochlorite (5000 ppm) and rinsed with deionized water to remove any residues between uses and prevent contamination. Throughout the process, sterile gloves were worn for sample collection and handling. All filtered membrane samples were folded, wrapped in sterile aluminum foil, and stored at −80 °C on the survey vessel until transported back to the laboratory for analysis.

DNA extraction from the water samples was performed using the Qiagen DNeasy Tissue and Blood DNA extraction kit (Qiagen, Venlo, The Netherlands) based on the manufacturer’s protocol. After completing the genomic DNA extraction, the extracted DNA was quantified using a UV spectrophotometer and stored at −80 °C. The total sample size obtained was 198.

### 2.2. PCR Amplification and High-Throughput Sequencing

We randomly selected representative samples for preliminary experiments to ensure that the majority of samples could amplify products of appropriate concentrations at the lowest cycle numbers. The target PCR fragment was a partial sequence of the 12S rRNA gene. The primer sequences used were as follows: MiFish-U-F5′-barcode-GTTGGTAAATCTCGTGCCAGC-3′ and MiFish-U-R5′-barcode-CATAGTGGGGTATCTAATCCTAGTTTG-3′ [18], which target the fish 12S rDNA region amplifying a ~180 bp region, to identify fish species. A barcode is an eight-base sequence used to distinguish different samples after multiplexed sequencing. The PCR conditions were as follows: initial denaturation at 95 °C for 5 min, followed by 30 cycles of denaturation at 95 °C for 30 s, annealing at 55 °C for 30 s, extension at 72 °C for 20 s, and final extension at 72 °C for 5 min. We extracted amplicons from 2% agarose gels and purified them using the AxyPrep DNA Gel Extraction Kit (Axygen Biosciences, Union City, CA, USA) according to the manufacturer’s instructions.

We quantified purified PCR products using Qubit^®^3.0 (Life Invitrogen, Waltham, MA, USA), and every twenty-four amplicons whose barcodes were different were mixed in equal amounts. We used the pooled DNA product to construct a Pair-End library using next^®^ultra™dna library prep kit (NEB, Ipswich, MA, USA) according to the manufacturer’s instructions. We then performed paired-end sequencing of the amplicon library on the Illumina MiSeq PE 250 platform (Shanghai Biozeron, Shanghai, China).

The raw reads were filtered by Trimmomatic [22] to remove lower-quality reads, such as those less than 50 bp in length and those with a tail quality of less than 20 bases. Pairs of reads were merged into a sequence using FLASH (v1.2.10) [23] based on the overlap (with a minimum length of 10 bp) relationship between raw reads. Chimeras were removed by a combination of de novo and reference sequences using Usearch (v6.1.544) software and the gold database, and primers were removed using Cutadapt (v4.0) (https://cutadapt.readthedocs.io/ (accessed on 1 May 2024)). QIIME2 (2020.11) software was utilized for annotation screening and further analysis. Sequence denoising was performed following the QIIME2 DADA2 (v1.18.0) analysis process. The dereplicated sequence generated through dada2 quality control was considered an amplicon sequence variant (ASV) with default parameters [24,25]. Taxonomic classification of representative sequences from each ASV was performed against the National Center for Biotechnology Information (NCBI) GenBank database and Mitofish databases (http://mitofish.aori.u-tokyo.ac.jp/ (accessed on 1 May 2024)). Significant hits were defined as those with e-values ≤ 1 × 10^−10^, calculated using the BLASTn algorithm (v2.2.30). Marine fish species were identified from the taxonomic annotation results using the FishBase database (www.fishbase.org (accessed on 1 May 2024)), followed by removal of potential false positives. Specifically, species with read counts ≤ 5 in any individual sample were classified as false positives and excluded from downstream analyses by setting their abundance values to zero. Subsequent analyses were performed on this filtered dataset.

Statistical analyses were performed using GNU package R 4.3.2 [26]: Generalized Linear Model (GLM) with a Poisson distribution to compare differences in species richness among the 3 layers, with layer as the independent factor; Pearson correlation to quantify linear relationships between species relative abundances; Pearson’s chi-square tests for categorical data comparisons; and spatial regression modeling using spatialreg package to assess associations between geographic coordinates (latitude, longitude) and diversity indices. Additionally, the rarefaction analysis based on Mothur was conducted to reveal the α-diversity indices [27], and Nonmetric Multidimensional Scaling Analysis (NMDS) was performed using the vegan and ggplot2 packages in R 4.3.2.

## 3. Results

### 3.1. High-Throughput Sequencing

Due to the concentration of some samples being too low, we successfully extracted eDNA from 176/198 samples (88.9% success rate) (90 surface water samples, 54 middle water samples, and 32 deep water samples), and we performed high-throughput sequencing. The distribution of sampling stations for all successful high-throughput sequencing water samples is shown in Figure 1. After quality control and screening, a total of 10,919,908 sequences were obtained from the 176 samples. The number of sequences in each sample ranged from 48,754 to 69,907, with an average of 62,044 ± 4692 (Supplementary Materials: Table S1).

### 3.2. Annotation Species Statistics

After quality control and de-duplication, the number of ASVs generated by sequences was 202,230, of which 65,794 ASVs were annotated to species. The initial number of fish species annotated was 296. After further removal of freshwater fish species and false positive species, the final number of annotated marine fish species is 98. These species belong to one phylum (Chordata), two classes (Actinopterygii and Chondrichthyes), 20 orders, 35 families, and 60 genera (Table 1).

**Figure 2:**
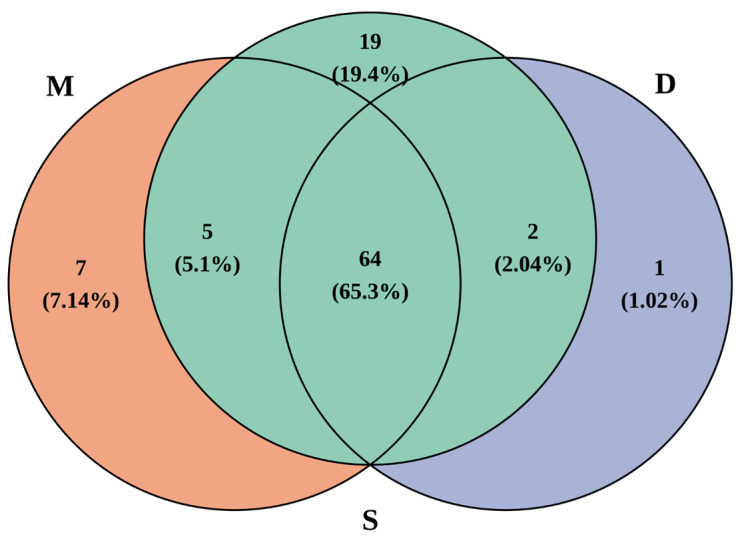
Venn diagram of species detected in different water layers (S represents surface water, M represents middle water, D represents deep water).

**Figure 3:**
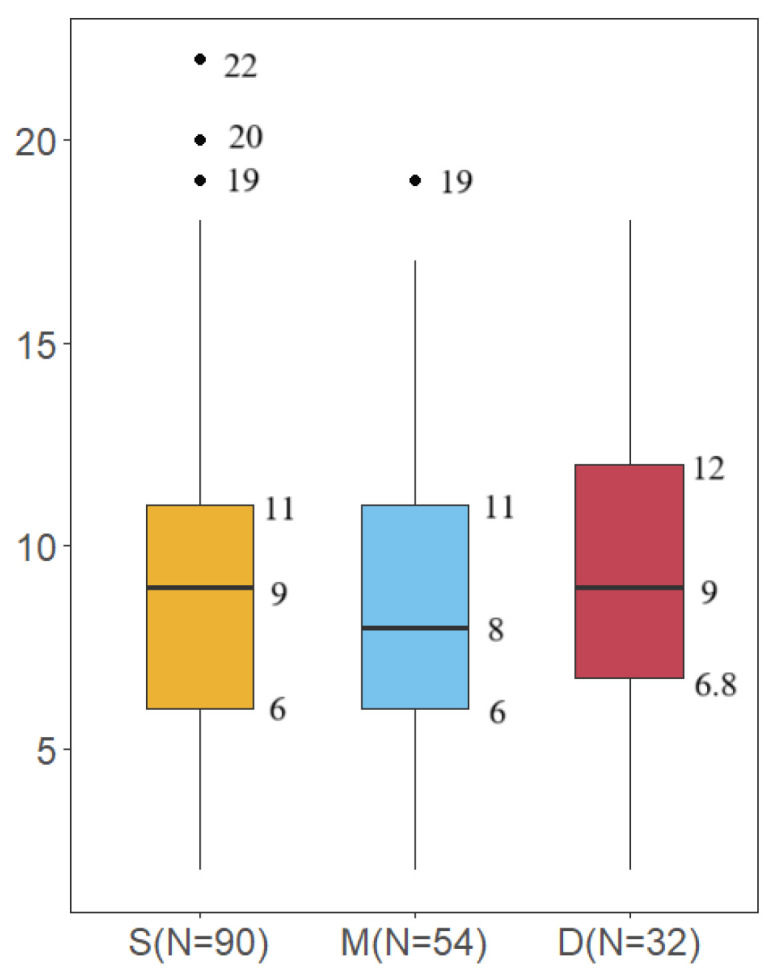
The number of species observed in samples from different water layers (S represents surface water, M represents middle water, D represents deep water).

A total of 90 species were detected in the surface water, 76 species in the middle water, and 67 species in the deep water (Figure 2). In the surface water samples, the number of species detected in each sample ranged from 2 to 22, with an average of 9.19 ± 4.14. In the middle water samples, it ranged from 2 to 19, with an average of 9.81 ± 4.89, and in the deep water samples, it ranged from 2 to 18, with an average of 9.38 ± 4.73 (Figure 3).

**Figure 4:**
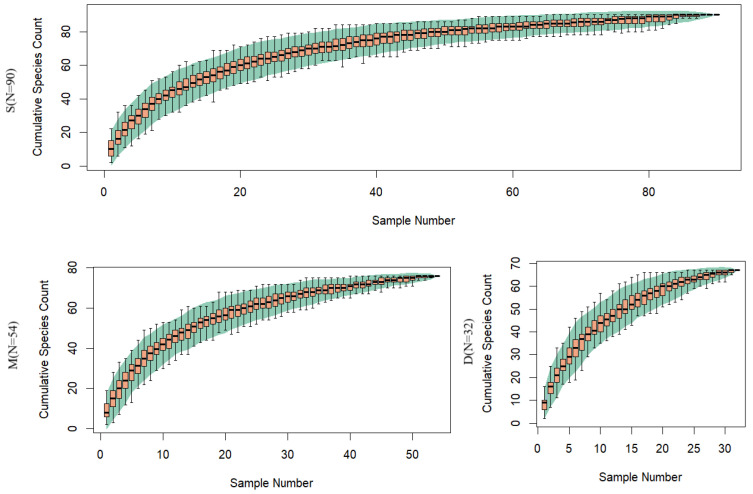
The species accumulation curves based on different surface water samples (S represents surface water, M represents middle water, D represents deep water).

The species accumulation curves (Figure 4) based on water samples from different depths show that the curve for the surface layer tends to level off, while the curves for the middle and deep layers are still on the rise with the current sampling effort, especially for the deep layer samples.

### 3.3. Comparison of Species Diversity Across Different Water Layers

**Figure 5:**
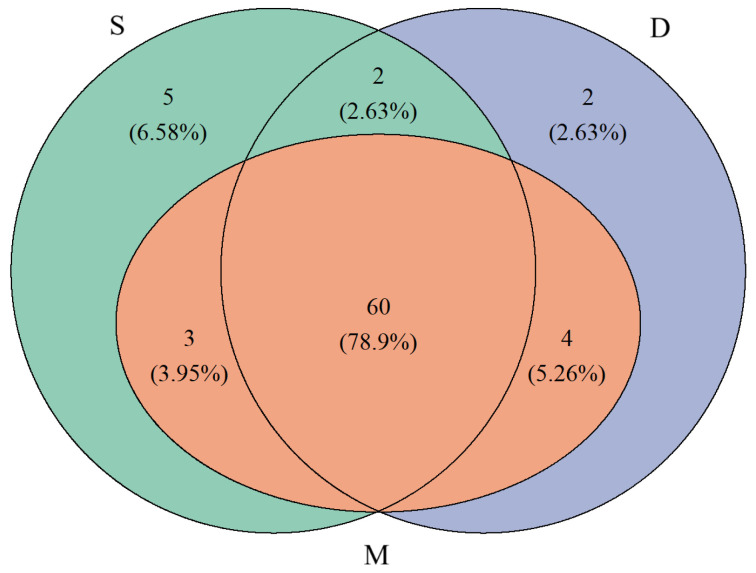
Venn diagram of species detected in different water layers of 28 stations (S indicates surface water; M indicates middle water; D indicates deep water).

**Figure 6:**
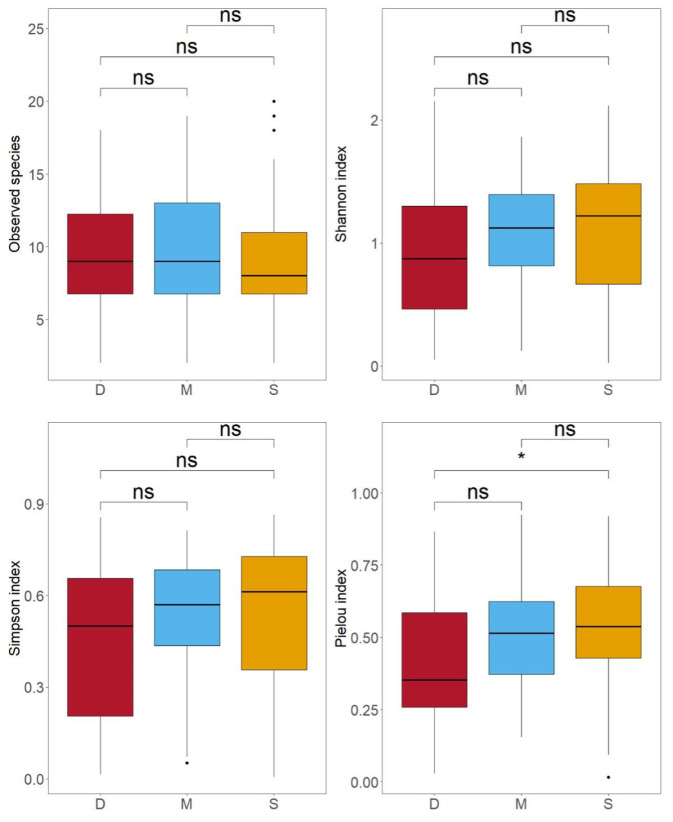
The distributions of observed species, Shannon index, Simpson index, and Pielou index for different water layers of 28 stations (S indicates surface water; M indicates middle water; D indicates deep water; ns indicates no significant difference; an asterisk (*) indicates a significant difference with *p* < 0.05).

**Figure 7:**
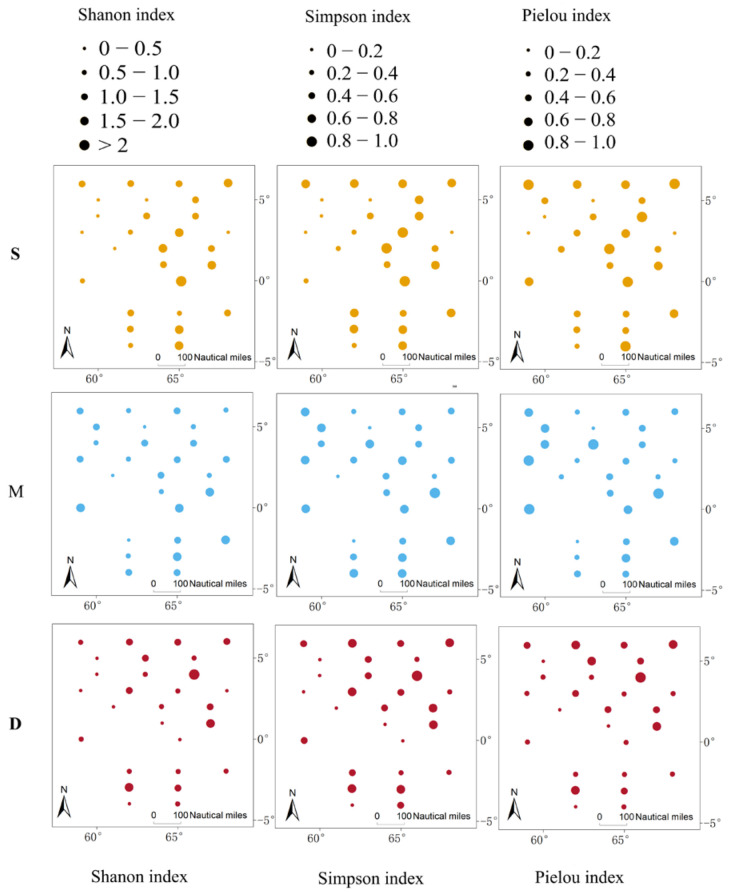
The Shannon, Simpson, and Pielou index for 28 stations (S indicates surface water; M indicates middle water; D indicates deep water).

To compare the differences in species composition and diversity among samples from different water layers, we separately analyzed data from the 28 stations where we had collected surface, middle, and deep water samples (yellow dots in Figure 1). In these 28 stations, a total of 70 species were observed in the surface water, slightly higher than the 67 species in the middle water and 68 species in the deep water (Figure 5). All species found in the middle water layer could also be detected in the surface and deep layers (Figure 5). However, Poisson GLM showed that the difference in the number of species detected in the samples from the three water layers is not statistically significant (Figure 6). In terms of α-diversity, as the water depth increased, all diversity indices (Shannon index, Simpson index, and Pielou index) showed decreasing trends (Figure 6). However, except for the Pielou index in the surface water, which was significantly higher than in the deep water (*p* < 0.05), no significant differences were observed in the other diversity indices among the different layers. The diversity indices for 28 stations are shown in Figure 7. The spatial regression analyses failed to reveal significant associations between any diversity indices and geographic coordinates (*p* > 0.1). Within the sampling area, the indices do not exhibit any particular trend with changes in latitude and longitude.

### 3.4. Relative Abundance of Species

**Figure 8:**
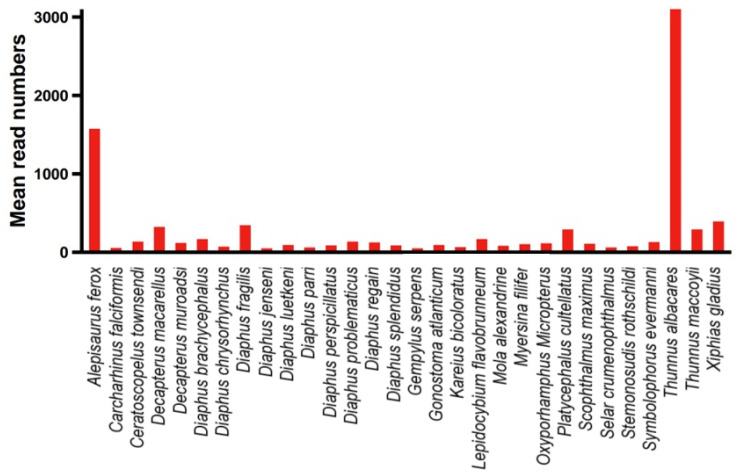
Top 30 species ranked by average reads count in the samples.

**Figure 9:**
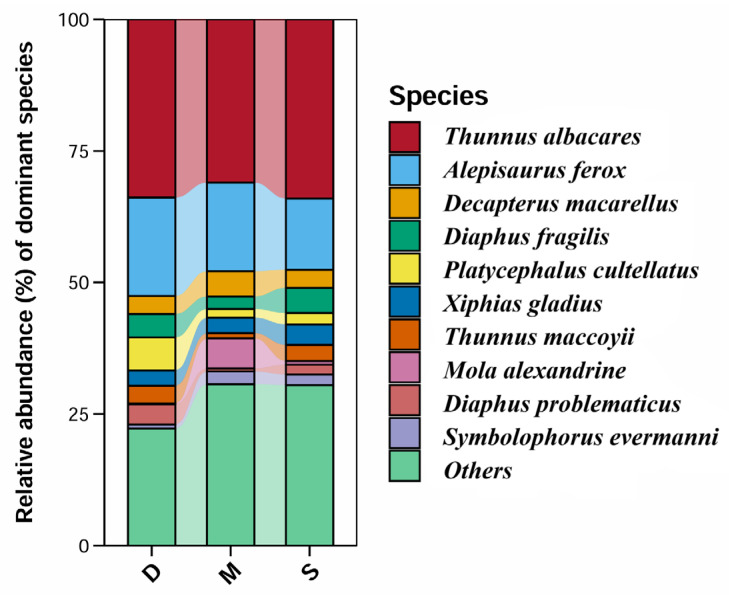
Relative abundance of dominant species in different water layers.

**Figure 10:**
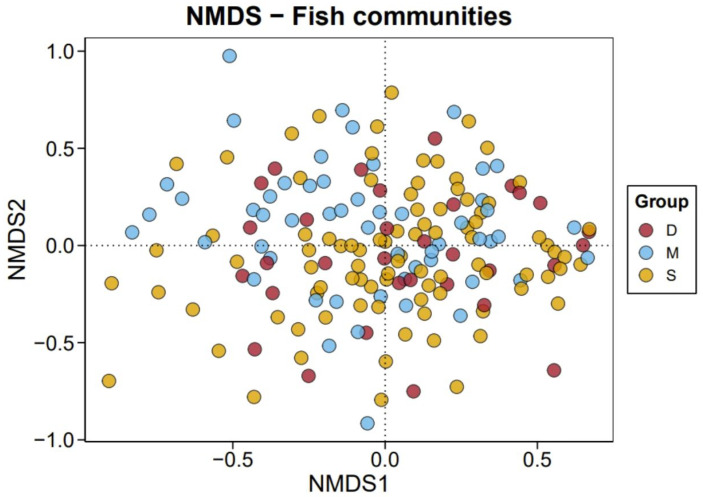
Nonmetric Multidimensional Scaling Analysis (NMDS) results of all samples.

**Figure 11:**
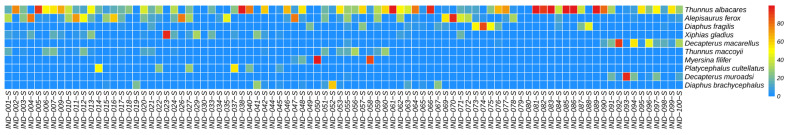
Heatmap of fish community in surface water samples.

**Figure 12:**
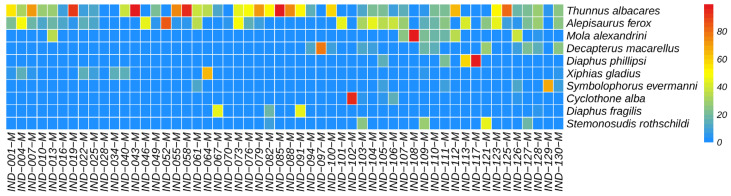
Heatmap of fish community in middle water samples.

**Figure 13:**
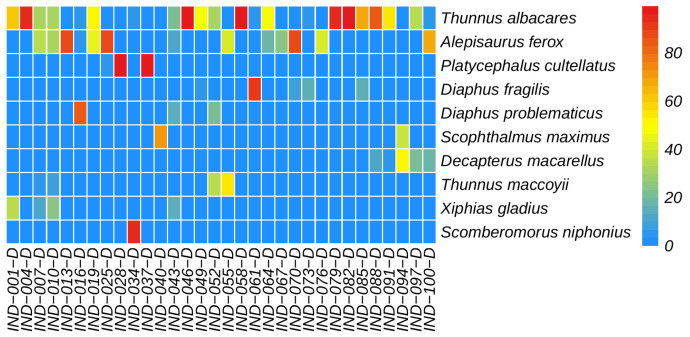
Heatmap of fish community in deep water samples.

**Figure 14:**
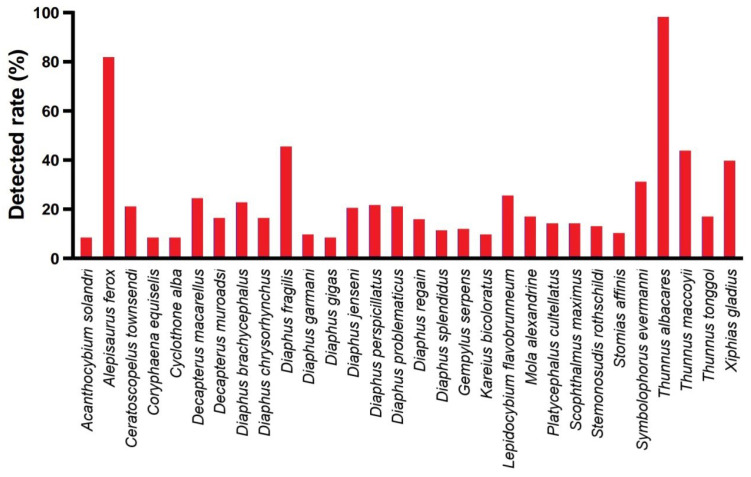
The top 30 species with the highest detection rates among all samples.

Among all monitored fish, the dominant class was the Actinopterygii fish. Among the monitored fish orders, the dominant was Scombriformes (40.72%), with the next most dominant orders being Aulopiformes (18.05%) and Myctophiformes (17.72%). At the family level, the dominant was Scombridae (38.03%), followed by Myctophidae (17.72%) and Alepisauridae (17.20%). The top 30 species ranked by abundance (average reads count) are shown in Figure 8, with the highest-ranked species being the *Thunnus albacares* (Bonnaterre, 1788), followed by the *Alepisaurus ferox* (Lowe, 1833). *T. albacares* and *A. ferox* together account for about 50% of the reads. Other abundant species include: *Xiphias gladius* (Linnaeus, 1758), *Diaphus fragilis* (Tåning, 1928), *Decapterus macarellus* (Cuvier, 1833), *Thunnus maccoyii* (Castelnau, 1872), and *Platycephalus cultellatus* (Richardson, 1846). Additionally, the results of a chi-square test showed that there was no significant difference in species composition across different water layers, with the relative abundance rankings of species being relatively consistent (Figure 9). Furthermore, the NMDS analysis also demonstrated similar results, showing no significant intergroup differentiation (Figure 10). However, species composition differs greatly between sampling stations (Figure 11, Figure 12 and Figure 13). There was generally no significant correlation between the abundances of different species. We also recorded the detection rate of each species across all samples, with the top 30 species shown in Figure 14. The relative abundance of *T. albacares*, the most abundant species, was detected in all samples, while the second-ranked *A. ferox* was detected in approximately 82% of the samples.

## 4. Discussion

### 4.1. Application Potential of eDNA Metabarcoding in Pelagic Fish Diversity Survey

Using eDNA metabarcoding for aquatic species diversity surveys is highly attractive, and its potential has been demonstrated in numerous studies [28]. Previous fish eDNA detection studies have often focused on freshwater or nearshore regions. The challenges of pelagic research lie in the difficulty of sampling, and compared to nearshore and freshwater areas, pelagic regions lack sufficient fishery catch data for comparison and support [29]. However, this also highlights the significant importance of conducting fish species diversity surveys in the pelagic zone using eDNA.

In this study, eDNA metabarcoding was applied to assess species diversity in the pelagic zone of the Western Indian Ocean. While evaluating the fish composition of the region, we also explored the potential application of eDNA metabarcoding for fish detection in pelagic areas. Even after removing species with low copy numbers, which were considered false positives, we detected 98 fish species. Although the pelagic region was generally considered to have fewer species due to its simple environment and limited habitats and resources, this study still achieved a considerable number of species observations compared to previous studies [20,28,30]. The main reason for this was the large sample size in this study. However, the number of species identified from a single sample was quite low, typically ranging from 2 to 22 species (Figure 3). There are several possible reasons for the low number of species identified in a single sample, including the relatively low number of species in the pelagic region, the low concentration of eDNA, and the incompleteness of the database. Another possible reason is that the higher water temperature in the tropical region increases the degradation rate of eDNA [31]. In the current study, the water filtration volume for each sample was 1 L, and we speculate that this volume may not be sufficient. This also led to the failure of DNA extraction in some samples due to low eDNA concentration. In future eDNA studies in tropical pelagic regions, increasing the volume of water filtration will be necessary. Furthermore, a larger sample size is essential, and this conclusion is further supported by the species accumulation curve (Figure 4).

Due to differences in sample size, the number of species obtained from surface samples (90) in this study was greater than that from the middle water (76), which was in turn greater than that from the deep water (67). To objectively compare the differences in species composition and species diversity across different water layers, we separately analyzed the 28 sampling stations that collected surface, middle water, and deep water. When the sample sizes were equal, the number of species identified from different water layer samples showed no significant differences (Figure 6). Furthermore, there were 5 species unique to the surface water, 2 species unique to the deep water, and all species identified in the mid-water samples could also be found in the surface and deep water (Figure 5). Apart from the number of species, the Shannon index, Simpson index, and Pielou index for different water layers were very similar (Figure 6). This indicated that, within the 300 m depth range covered in this study, there was no significant difference in species diversity. The comparison of different water layer samples suggests that collecting only surface and deep water samples is sufficient for species identification, and middle water samples can be omitted. If the goal is to further reduce sampling costs, collecting only surface water samples would cover the vast majority of species and diversity information. It is also worth noting that no clear pattern was observed in the diversity indices across different sampling stations, indicating that species identification at different stations has a significant degree of randomness. The primary source of randomness may stem from the fact that eDNA is not uniformly distributed within water bodies but exists as particles of varying sizes [32]. Additionally, variations in sampling timing [33] and the impacts of hydrodynamic processes such as water movement and diffusion [34] may contribute to this observed randomness. To address these challenges, in addition to the previously recommended strategy of increasing sample size, implementing standardized sampling protocols across large spatial scales while comprehensively accounting for environmental variables and hydrodynamic influences will be crucial for enhancing the accuracy and reliability of future eDNA-based studies.

### 4.2. Detected Relative Abundance of Species

Due to the lack of trawl survey data in the Western Indian Ocean, this study had to rely on historical catch data for comparison. The fish families with the highest relative abundance in this study included Scombridae, Myctophidae, and Alepisauridae. According to FAO statistics (https://www.fao.org/fishery/en/fishstat (accessed on 5 June 2024)), the Scombridae family accounted for the highest catch in the Western Indian Ocean, making up 50% of the total catch. In this study, the relative abundance of Scombridae was also quite high (38.02%). Other fish species with higher catch rates in the Western Indian Ocean included Clupeidae, Carangidae, and Serranidae. Clupeidae and Carangidae were less abundant, while Serranidae was not detected. The suspected reason for this is that the sampling area in this study is located in the oceanic region, which is different from the habitats of these species.

Correspondingly, many species within two of the three orders with the highest relative abundance detected in this study (Aulopiformes and Myctophiformes) lack corresponding fishery catch data. Beyond differences in survey areas, another more significant reason lies in the fact that traditional fishery surveys focus exclusively on commercially valuable species (i.e., landed catches for sale), while Aulopiformes and Myctophiformes fish are often considered fishery discards. This also demonstrates an advantage of eDNA methodology over conventional fishery surveys.

According to FAO statistics, the most caught species in the Western Indian Ocean are *Sardinella longiceps* (Valenciennes, 1847), *Katsuwonus pelamis* (Linnaeus, 1758), and *T. albacares*. The *S. longiceps* is considered a small pelagic fish confined to the continental shelf waters [35], which is why it was not found in this study. On the other hand, both *K. pelamis* and *T. albacares* were monitored in this study, with *T. albacares* being the most abundant species, ranking highest in relative abundance. Moreover, this species was found in all of the samples. *T. albacares* is highly mobile and is considered to be widely distributed across tropical and subtropical regions of the Atlantic, Pacific, and Indian Oceans [36]. Meanwhile, it was an important component of global fisheries, comprising 31% of the total catches of major commercial tuna species [2]. These reasons explain why this species was widely present and had extremely high abundance in the samples of this study. Surprisingly, although *K. pelamis* was detected in this survey, its relative abundance was relatively low, especially when compared to its extremely high catch yield. This reflects that an eDNA-based survey still cannot fully meet the needs of assessing fish relative abundance. For example, errors introduced by differences in PCR amplification efficiency of different sequence types in eDNA samples are unavoidable. Sequencing read counts cannot accurately represent the relative abundance of eDNA from different species in the sample.

Other economically important fish species with relatively high abundance detected in this study include *X. gladius*, *D. macarellus*, *T. maccoyii*, and *P. cultellatus.* However, there is some difference between the economic species types and their relative abundance rankings found in this study compared to the fishery catch data. The potential reasons for this discrepancy are varied, with the primary reason being that the sampling area in this study is limited to the oceanic region of the Western Indian Ocean.

### 4.3. The Improvement Direction of Methodology

Although eDNA-based fish surveys have been widely applied, this method still has several limitations. For example, this study identified some species that should not have been present in the Indian Ocean (such as *Scophthalmus maximus* (Linnaeus, 1758)). In addition to potential false positives, the removal of low-copy-number species in this study may lead to the loss of rare species (false negatives). Establishing a comprehensive and targeted barcode database is an important solution to this issue. Although this task is challenging, it is still necessary to enhance the practicality of eDNA metabarcoding [37].

In this study, we only used a pair of universal primers as the research tool, while the selection of universal primers can also impact taxonomic coverage. Considering that tropical oceans are rich in fish diversity [37], the species detection rate using a single primer might be unsatisfactory. To address these limitations, using multiple sets of universal primers (e.g., 12S + COI), along with validating and supplementing results from different primers, is an effective way to improve the reliability of eDNA survey results [38]. Additionally, environmental factors that affect eDNA persistence, such as ocean mixing [39], temperature, and depth gradients [40,41], were not analyzed in this study. In future research, considering environmental factors and understanding eDNA behavior in tropical aquatic environments, as well as standardizing analytical procedures, are also ways to enhance the reliability of marine eDNA studies [37,40,41].

## 5. Conclusions

This study is the first to investigate the fish diversity in the Western Indian Ocean’s pelagic zone using eDNA metabarcoding, detecting a total of 98 fish species, including many important economic species. The study found that within a depth range of 300 m, the species and diversity indices identified from eDNA samples at different water layers were similar. The species composition and relative abundance of economic species observed in this study differed somewhat from FAO fishery catch statistics, a result that was anticipated. The results of this study provide a new perspective on species diversity in the Western Indian Ocean and suggest that eDNA is an important complement to traditional survey methods to monitor fish biodiversity in the oceanic region.

## Figures and Tables

**Table 1:** The 98 species of fish detected using eDNA in the Western Indian Ocean and their classification information.

Class	Order	Family	Species	Authority
Actinopterygii	Acanthuriformes	Acanthuridae	*Acanthurus leucosternon*	Bennett, 1833
Actinopterygii	Actinopteri_norank	Pseudochromidae	*Pseudoplesiops revellei*	Schultz, 1953
Actinopterygii	Actinopteri_norank	Sciaenidae	*Johnius trewavasae*	Sasaki, 1992
Actinopterygii	Anguilliformes	Nemichthyidae	*Nemichthys scolopaceus*	Richardson, 1848
Actinopterygii	Anguilliformes	Ophichthidae	*Muraenichthys gymnopterus*	Bleeker, 1853
Actinopterygii	Aulopiformes	Alepisauridae	*Alepisaurus ferox*	Lowe, 1833
Actinopterygii	Aulopiformes	Chlorophthalmidae	*Chlorophthalmus pectoralis*	Okamura & Doi, 1984
Actinopterygii	Aulopiformes	Paralepididae	*Lestrolepis intermedia*	Poey, 1868
Actinopterygii	Aulopiformes	Paralepididae	*Stemonosudis miscella*	Ege, 1933
Actinopterygii	Aulopiformes	Paralepididae	*Stemonosudis rothschildi*	Richards, 1967
Actinopterygii	Beloniformes	Exocoetidae	*Cheilopogon abei*	Parin, 1996
Actinopterygii	Beloniformes	Exocoetidae	*Cheilopogon atrisignis*	Günther, 1866
Actinopterygii	Beloniformes	Exocoetidae	*Cheilopogon intermedius*	Parin, 1961
Actinopterygii	Beloniformes	Exocoetidae	*Cheilopogon pinnatibarbatus*	Bennett, 1831
Actinopterygii	Beloniformes	Exocoetidae	*Exocoetus monocirrhus*	Richardson, 1846
Actinopterygii	Beloniformes	Exocoetidae	*Hirundichthys affinis*	Günther, 1866
Actinopterygii	Beloniformes	Exocoetidae	*Hirundichthys rondeletii*	Valenciennes, 1847
Actinopterygii	Beloniformes	Hemiramphidae	*Hemiramphus convexus*	Weber, 1914
Actinopterygii	Beloniformes	Hemiramphidae	*Oxyporhamphus micropterus*	Valenciennes, 1847
Actinopterygii	Carangiformes	Carangidae	*Decapterus macarellus*	Cuvier, 1833
Actinopterygii	Carangiformes	Carangidae	*Decapterus muroadsi*	Temminck & Schlegel, 1844
Actinopterygii	Carangiformes	Carangidae	*Elagatis bipinnulata*	Quoy & Gaimard, 1825
Actinopterygii	Carangiformes	Carangidae	*Selar crumenophthalmus*	Bloch, 1793
Actinopterygii	Carangiformes	Coryphaenidae	*Coryphaena equiselis*	Linnaeus, 1758
Actinopterygii	Carangiformes	Coryphaenidae	*Coryphaena hippurus*	Linnaeus, 1758
Actinopterygii	Chaetodontiformes	Leiognathidae	*Nuchequula nuchalis*	Temminck & Schlegel, 1845
Actinopterygii	Clupeiformes	Clupeidae	*Clupanodon thrissa*	Linnaeus, 1758
Actinopterygii	Clupeiformes	Engraulidae	*Coilia nasus*	Temminck & Schlegel, 1846
Actinopterygii	Gobiiformes	Gobiidae	*Acanthogobius flavimanus*	Temminck & Schlegel, 1845
Actinopterygii	Gobiiformes	Gobiidae	*Chaeturichthys stigmatias*	Richardson, 1844
Actinopterygii	Gobiiformes	Gobiidae	*Myersina filifer*	Valenciennes, 1837
Actinopterygii	Holocentriformes	Holocentridae	*Myripristis pralinia*	Cuvier, 1829
Actinopterygii	Istiophoriformes	Istiophoridae	*Tetrapturus angustirostris*	Tanaka, 1915
Actinopterygii	Istiophoriformes	Xiphiidae	*Xiphias gladius*	Linnaeus, 1758
Actinopterygii	Lampriformes	Regalecidae	*Regalecus glesne*	Ascanius, 1772
Actinopterygii	Mugiliformes	Mugilidae	*Planiliza haematocheilus*	Temminck & Schlegel, 1845
Actinopterygii	Mugiliformes	Mugilidae	*Planiliza macrolepis*	Smith, 1846
Actinopterygii	Myctophiformes	Myctophidae	*Bolinichthys longipes*	Brauer, 1906
Actinopterygii	Myctophiformes	Myctophidae	*Ceratoscopelus townsendi*	Eigenmann & Eigenmann, 1889
Actinopterygii	Myctophiformes	Myctophidae	*Ceratoscopelus warmingii*	Lütken, 1892
Actinopterygii	Myctophiformes	Myctophidae	*Diaphus brachycephalus*	Tåning, 1928
Actinopterygii	Myctophiformes	Myctophidae	*Diaphus chrysorhynchus*	Gilbert & Cramer, 1897
Actinopterygii	Myctophiformes	Myctophidae	*Diaphus fragilis*	Tåning, 1928
Actinopterygii	Myctophiformes	Myctophidae	*Diaphus fulgens*	Brauer, 1904
Actinopterygii	Myctophiformes	Myctophidae	*Diaphus garmani*	Gilbert, 1906
Actinopterygii	Myctophiformes	Myctophidae	*Diaphus gigas*	Gilbert, 1913
Actinopterygii	Myctophiformes	Myctophidae	*Diaphus jenseni*	Tåning, 1932
Actinopterygii	Myctophiformes	Myctophidae	*Diaphus luetkeni*	Brauer, 1904
Actinopterygii	Myctophiformes	Myctophidae	*Diaphus parri*	Tåning, 1932
Actinopterygii	Myctophiformes	Myctophidae	*Diaphus perspicillatus*	Ogilby, 1898
Actinopterygii	Myctophiformes	Myctophidae	*Diaphus phillipsi*	Fowler, 1934
Actinopterygii	Myctophiformes	Myctophidae	*Diaphus problematicus*	Parr, 1928
Actinopterygii	Myctophiformes	Myctophidae	*Diaphus regani*	Tåning, 1932
Actinopterygii	Myctophiformes	Myctophidae	*Diaphus splendidus*	Brauer, 1904
Actinopterygii	Myctophiformes	Myctophidae	*Diaphus suborbitalis*	Weber, 1913
Actinopterygii	Myctophiformes	Myctophidae	*Diaphus termophilus*	Tåning, 1928
Actinopterygii	Myctophiformes	Myctophidae	*Lampadena anomala*	Parr, 1928
Actinopterygii	Myctophiformes	Myctophidae	*Lampadena atlantica*	Maul, 1969
Actinopterygii	Myctophiformes	Myctophidae	*Lampadena luminosa*	Garman, 1899
Actinopterygii	Myctophiformes	Myctophidae	*Lampadena yaquinae*	Coleman & Nafpaktitis, 1972
Actinopterygii	Myctophiformes	Myctophidae	*Myctophum lychnobium*	Bolin, 1946
Actinopterygii	Myctophiformes	Myctophidae	*Notolychnus valdiviae*	Brauer, 1904
Actinopterygii	Myctophiformes	Myctophidae	*Symbolophorus californiensis*	Eigenmann & Eigenmann, 1889
Actinopterygii	Myctophiformes	Myctophidae	*Symbolophorus evermanni*	Gilbert, 1905
Actinopterygii	Perciformes	Platycephalidae	*Platycephalus cultellatus*	Richardson, 1846
Actinopterygii	Perciformes	Sebastidae	*Sebastes constellatus*	Jordan & Gilbert, 1880
Actinopterygii	Perciformes	Sebastidae	*Sebastes goodei*	Eigenmann & Eigenmann, 1890
Actinopterygii	Perciformes	Sebastidae	*Sebastes koreanus*	Kim & Lee, 1994
Actinopterygii	Perciformes	Sebastidae	*Sebastes melanosema*	Lea, 1911
Actinopterygii	Perciformes	Sebastidae	*Sebastes rosenblatti*	Chen, 1971
Actinopterygii	Pleuronectiformes	Pleuronectidae	*Kareius bicoloratus*	Basilewsky, 1855
Actinopterygii	Pleuronectiformes	Pleuronectidae	*Pleuronectes platessa*	Linnaeus, 1758
Actinopterygii	Pleuronectiformes	Scophthalmidae	*Scophthalmus maximus*	Walbaum, 1792
Actinopterygii	Scombriformes	Bramidae	*Brama dussumieri*	Cuvier, 1831
Actinopterygii	Scombriformes	Gempylidae	*Gempylus serpens*	Cuvier, 1829
Actinopterygii	Scombriformes	Gempylidae	*Lepidocybium flavobrunneum*	Smith, 1849
Actinopterygii	Scombriformes	Nomeidae	*Psenes pellucidus*	Lütken, 1880
Actinopterygii	Scombriformes	Scombridae	*Acanthocybium solandri*	Cuvier, 1832
Actinopterygii	Scombriformes	Scombridae	*Allothunnus fallai*	Serventy, 1948
Actinopterygii	Scombriformes	Scombridae	*Auxis rochei*	Risso, 1810
Actinopterygii	Scombriformes	Scombridae	*Auxis thazard*	Lacepède, 1800
Actinopterygii	Scombriformes	Scombridae	*Katsuwonus pelamis*	Linnaeus, 1758
Actinopterygii	Scombriformes	Scombridae	*Scomberomorus niphonius*	Cuvier, 1832
Actinopterygii	Scombriformes	Scombridae	*Thunnus alalunga*	Bonnaterre, 1788
Actinopterygii	Scombriformes	Scombridae	*Thunnus albacares*	Bonnaterre, 1788
Actinopterygii	Scombriformes	Scombridae	*Thunnus maccoyii*	Castelnau, 1872
Actinopterygii	Scombriformes	Scombridae	*Thunnus orientalis*	Temminck & Schlegel, 1844
Actinopterygii	Scombriformes	Scombridae	*Thunnus thynnus*	Linnaeus, 1758
Actinopterygii	Scombriformes	Scombridae	*Thunnus tonggol*	Bleeker, 1851
Actinopterygii	Scombriformes	Scombrolabracidae	*Scombrolabrax heterolepis*	Roule, 1921
Actinopterygii	Stomiiformes	Gonostomatidae	*Cyclothone alba*	Brauer, 1906
Actinopterygii	Stomiiformes	Gonostomatidae	*Gonostoma atlanticum*	Norman, 1930
Actinopterygii	Stomiiformes	Stomiidae	*Borostomias pacificus*	Imai, 1941
Actinopterygii	Stomiiformes	Stomiidae	*Stomias affinis*	Günther, 1887
Actinopterygii	Tetraodontiformes	Molidae	*Mola alexandrini*	Ranzani, 1839
Chondrichthyes	Carcharhiniformes	Carcharhinidae	*Carcharhinus falciformis*	Müller & Henle, 1839
Chondrichthyes	Carcharhiniformes	Carcharhinidae	*Prionace glauca*	Linnaeus, 1758
Chondrichthyes	Myliobatiformes	Dasyatidae	*Pteroplatytrygon violacea*	Bonaparte, 1832

## Data Availability

The raw data supporting the conclusions of this article will be made available by the authors on request.

## Supplementary Materials

The following Supporting Information can be downloaded at: https://www.mdpi.com/article/10.3390/biology14091194/s1, Table S1: Data size and average sequence length of 176 samples.
