# Supplementary material for: Monitoring Fish Biodiversity in the Pelagic Zone of the Western Indian Ocean Using Environmental DNA Metabarcoding
Source: Biology (Basel). 2025 Sep 4;14(9):1194. doi: 10.3390/biology14091194 (PMC12467256; doi:10.3390/biology14091194)
Supplement: Supplementary file 1 [file biology-14-01194-s001.zip › biology-3762642-supplementary.pdf]

Table S1 Data size and average sequence length of 176 samples

Samples	Sequences	Average Length(bp)	Samples	Sequences	Average Length(bp)
IND-001-D	68348	171.23	IND-061-D	69517	170.25
IND-001-M	64374	171.89	IND-061-M	62005	173.21
IND-001-S	58188	170.32	IND-061-S	59523	172.4
IND-002-S	55076	170.58	IND-062-S	62540	173.67
IND-003-S	63056	170.16	IND-063-S	64625	169.79
IND-004-D	58077	169.9	IND-064-D	67433	170.64
IND-004-M	61349	169.92	IND-064-M	64840	175.18
IND-004-S	56182	170.33	IND-064-S	55792	186.01
IND-005-S	62386	170.18	IND-065-S	63338	169.48
IND-006-S	64423	171.67	IND-066-S	55521	172.06
IND-007-D	65013	173.02	IND-067-D	61308	173.55
IND-007-M	60664	173.95	IND-067-M	56286	175.7
IND-007-S	55943	169.93	IND-067-S	68229	172.63
IND-009-S	65941	177.25	IND-069-S	66448	176.35
IND-010-D	67316	172.13	IND-070-D	61913	175.84
IND-010-M	67937	171.52	IND-070-M	58126	171.08
IND-010-S	58686	172.93	IND-070-S	64042	170.61
IND-011-S	57578	170.3	IND-071-S	62264	173.78
IND-012-S	63769	172.28	IND-072-S	65623	171.91
IND-013-D	55400	172.94	IND-073-D	58021	174.52
IND-013-M	66655	172.01	IND-073-M	69783	177.45
IND-013-S	56374	174.51	IND-073-S	62333	168.83
IND-014-S	50125	175.95	IND-074-S	58384	179.02
IND-015-S	66264	171.55	IND-075-S	56769	174.38
IND-016-D	69256	179.75	IND-076-D	62888	170.23
IND-016-M	62538	179.27	IND-076-M	66978	169.13
IND-016-S	55828	170.28	IND-076-S	59551	177.76
IND-017-S	59183	180.64	IND-077-S	50014	172.57
IND-018-S	59368	173.25	IND-078-S	67905	173.19
IND-019-D	60490	182.07	IND-079-D	65881	172.56
IND-019-M	59999	171.14	IND-079-M	63701	173.66
IND-019-S	62039	169.64	IND-079-S	63555	172.64
IND-020-S	58644	170.35	IND-080-S	64263	175.92
IND-021-S	64695	171.25	IND-081-S	68658	178.78
IND-022-M	60696	169.51	IND-082-D	62543	169.72
IND-022-S	61218	195.71	IND-082-M	68633	178.33
IND-023-S	64910	170.75	IND-082-S	65300	173.07
IND-024-S	69907	185.23	IND-083-S	67535	170.95
IND-025-D	61487	173.71	IND-084-S	58726	170.13
IND-025-M	68972	182.06	IND-085-D	56860	170.47

IND-026-S	58492	175.15	IND-085-M	68986	189.7
IND-027-S	67554	175.84	IND-085-S	67439	172.12
IND-028-D	66254	189.64	IND-086-S	60882	169.54
IND-028-M	60174	174.18	IND-087-S	59950	169.88
IND-029-S	66345	169.1	IND-088-D	58470	171.24
IND-030-S	69546	174.62	IND-088-M	57431	174.35
IND-033-S	60461	167.95	IND-088-S	61093	173.06
IND-034-D	57979	170.31	IND-089-S	68096	171.16
IND-034-M	63547	181.31	IND-090-S	59744	167.82
IND-034-S	57157	177.43	IND-091-D	65547	173.32
IND-035-S	65076	173.32	IND-091-M	60069	171.88
IND-037-D	65308	177.75	IND-091-S	58010	170.04
IND-037-S	69558	170.91	IND-092-S	58316	182.87
IND-038-S	65083	171.71	IND-093-S	59409	171.3
IND-040-D	48754	172.94	IND-094-D	69617	174.4
IND-040-M	58944	171.13	IND-094-M	55988	170.21
IND-040-S	65447	181.72	IND-094-S	62374	174.25
IND-041-S	62964	174.7	IND-095-S	68053	168.95
IND-042-S	59026	171.04	IND-096-S	59286	173.19
IND-043-D	67631	169.89	IND-097-D	65807	169.2
IND-043-M	56762	167.9	IND-097-M	65625	170
IND-044-S	55369	170.44	IND-097-S	57086	171.27
IND-045-S	56626	175.03	IND-098-S	65390	172.08
IND-046-D	57303	171.51	IND-099-S	68320	172.17
IND-046-M	64081	175.19	IND-100-D	58751	170.2
IND-046-S	50281	174.25	IND-100-M	63824	172.73
IND-047-S	65872	172.05	IND-100-S	66996	172.46
IND-048-S	63210	180.39	IND-101-M	56468	170.18
IND-049-D	59635	190.56	IND-102-M	64109	171.84
IND-049-M	56741	185.63	IND-103-M	56070	173.98
IND-049-S	50065	174.61	IND-104-M	68815	172.06
IND-050-S	67154	182.44	IND-105-M	57121	170.79
IND-051-S	69721	170.4	IND-106-M	68451	168.2
IND-052-D	62280	170.72	IND-107-M	55675	170.3
IND-052-M	59895	172.73	IND-108-M	60225	168.48
IND-052-S	69027	171.17	IND-109-M	63220	172.79
IND-053-S	62873	173.55	IND-110-M	62440	171.62
IND-055-D	63100	173.19	IND-111-M	63080	172.6
IND-055-M	57547	181.82	IND-112-M	62231	171.73
IND-055-S	63860	170.09	IND-113-M	61598	169.42
IND-056-S	56896	172.1	IND-117-M	69202	171.59
IND-057-S	64355	172.57	IND-121-M	69874	170.76
IND-058-D	56850	169.41	IND-123-M	60540	171.26
IND-058-M	59944	176.26	IND-125-M	58398	175.4

IND-058-S	56876	173	IND-126-M	60811	173.63
IND-059-S	65738	173.38	IND-127-M	57273	170.75
IND-060-S	56172	178.3	IND-128-M	61757	171.86
